# Supplementary material for: Photoswitchable exceptional points derived from bound states in the continuum
Source: Light Sci Appl. 2025 Oct 28;14:377. doi: 10.1038/s41377-025-02036-0 (PMC12568961; doi:10.1038/s41377-025-02036-0)
Supplement: Supplementary file 1 — Supplementary Materials for Photoswitchable exceptional points derived from bound states in the continuum [file 41377_2025_2036_MOESM1_ESM.docx]

# Supplementary Materials for

**Photoswitchable exceptional points derived from** **bound states in the continuum**

Lei Wang^1^^†^, Hang Liu^1†^, Junwei Liu^1^, Aoxuan Liu^1^, Jialiang Huang^1^, Qiannan Li^1^, Hui Dai^1^, Caihong Zhang^1,2*^, Jingbo Wu^1,2^, Kebin Fan^1,2*^, Huabing Wang^1,2^, Biaobing Jin^1,2^, Jian Chen^1,2^, and Peiheng Wu^1,2^

^1^*Research Institute of Superconductor Electronics (RISE), Key Laboratory of Optoelectronic Devices and Systems with Extreme Performances of MOE, School of Electronic Science and Engineering, Nanjing University, Nanjing 210023, China*

^2^*Purple Mountain Laboratories, Nanjing 211111, China*

^†^*These authors contributed equally to this work*

*Correspondence should be addressed to:* *[chzhang@](mailto:chzhang@nju.edu.cn )**[nju.edu.cn](mailto:chzhang@nju.edu.cn ) and kebin.fan@nju.edu.cn.*

**Table of Contents**

**Section 1. Temporal coupled mode theory (TCMT)**

**Section 2. Effect of material losses**

**Section 3. Extraction of eigenfrequencies from the transmission spectrum**

**Section 4. Calculation of far-field polarization**

**Section 5. Sample fabrication**

**Section 6. Experimental characterization**

**References**

**Section 1. Temporal coupled mode theory (TCMT)**

TCMT can effectively describe the resonant response of the system with two or multiple modes. The temporal dynamics of the metasurface can be formulated as^1^

$\frac{d\boldsymbol{a}}{dt}=\left( -i\Omega_{0}-\Gamma\right)\boldsymbol{a}+K^{T}s_{+}$

$s_{-}=Cs_{+}+D\boldsymbol{a}$

where ***a*** is the resonance amplitude vector of the modes in the system with time-harmonic dependence of exp(-*iωt*). *i* is an imaginary unit satisfying $i^{2}=-1$. *Ω*_0_ and *Γ* are the matrices of the resonance frequencies and radiation loss rates consisting of *ω* and *γ*. To simplify the equation for facilitating the analysis, the intrinsic loss is not considered. *s_+_* and *s_-_* are the amplitude of the inputs and the outputs, which are associated with the *S*-parameters of the scattering matrix. *K* and *D* refer to the input and output coupling matrixes from the resonant modes to the ports, respectively. For lossless and reciprocal systems, *C* is generally considered to be unitary and symmetric matrix. Energy conservation and time-reversal symmetry imply that

$$D^{\dagger}D=2\Gamma$$

$$D=K$$

$$CD^{*}=-D$$

From the TCMT equations, we may obtain the scattering matrix *S* as

$S=\left( 1-iD\frac{1}{\omega-H_{\mathrm{eff}}}D^{\dagger} \right)C$

where *H*_eff_ = *H*_0_ − *iD*^†^*D* / 2 describes the effective Hamiltonian of the system taking into account radiative dissipation into the coupling channels. Consider a two-mode system, by solving the eigenvalues of the 2 × 2 matrix *H*_eff_, we can obtain the eigen complex frequencies

$\omega_{\mathrm{eig}1,2}=\frac{(\omega_{1}+\omega_{2})-i(\gamma_{1}+\gamma_{2})\pm\sqrt{\left[ (\omega_{1}-\omega_{2})-i(\gamma_{1}-\gamma_{2}) \right]^{2}+4(\kappa-i\sqrt{\gamma_{1}\gamma_{2}})^{2}}}{2}$

Under Friedrich-Wintgen (FW) condition$\kappa(\gamma_{1}-\gamma_{2})=\sqrt{\gamma_{1}\gamma_{2}}(\omega_{1}-\omega_{2})$, we can derive the eigenfrequency representation of the system as

$\omega_{\mathrm{eig}1}=\frac{(\omega_{1}+\omega_{2})}{2}-\frac{\kappa(\gamma_{1}+\gamma_{2})}{2\sqrt{\gamma_{1}\gamma_{2}}}$

$\omega_{eig2}=\frac{(\omega_{1}+\omega_{2})}{2}+\frac{\kappa(\gamma_{1}+\gamma_{2})}{2\sqrt{\gamma_{1}\gamma_{2}}}-i(\gamma_{1}+\gamma_{2})$

**
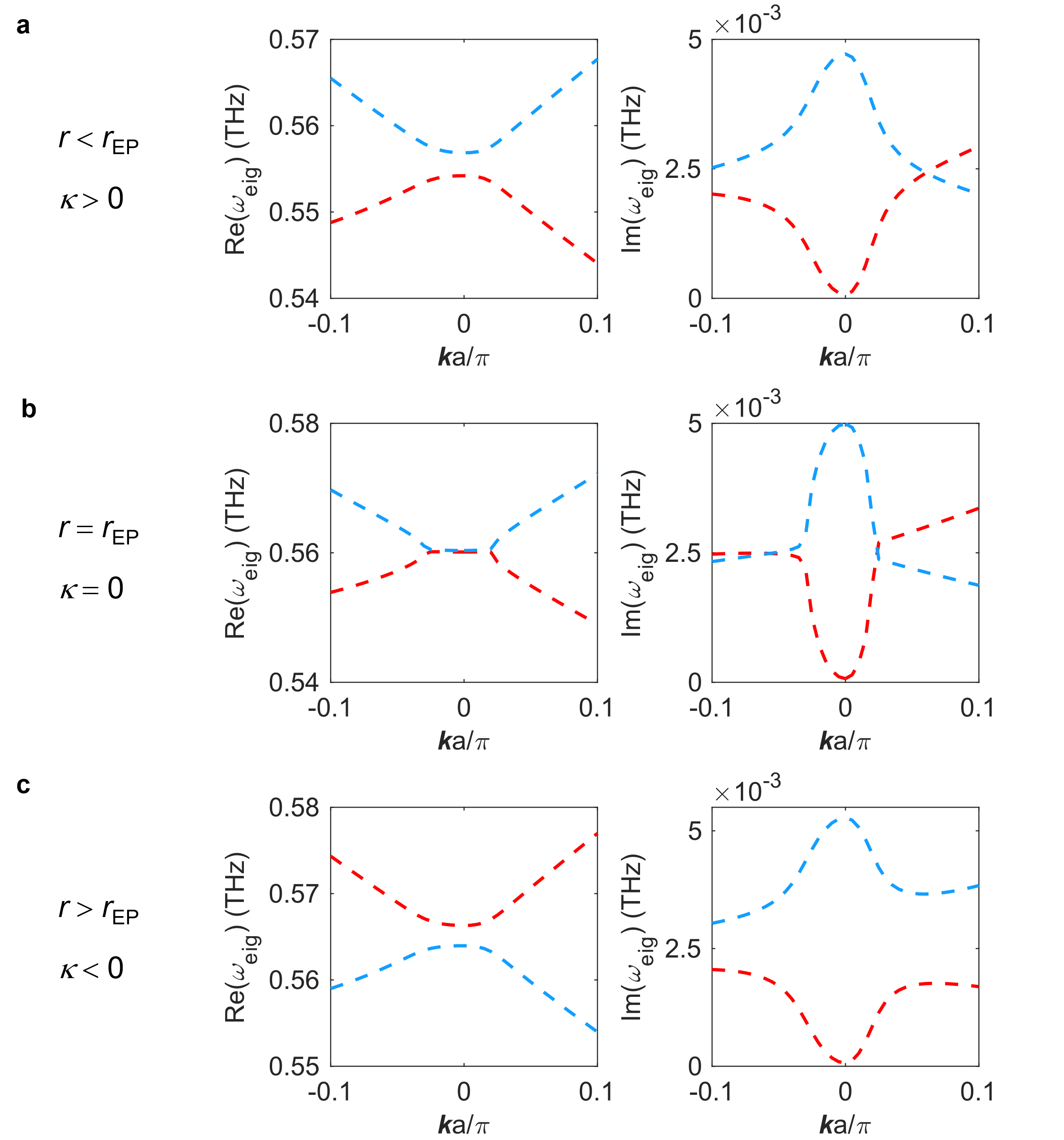
**

**Fig. S1.** Analysis of the complex eigenvalues of the metasurface under different near-coupling conditions. The simulation results include structures with different radii: **a**, *r* = 69.4 μm, **b,** *r* = 70.4 μm, and **c**, *r* = 71.4 μm, corresponding to *κ* > 0, *κ =* 0, and *κ* < 0, respectively. Each subplot displays both the real (left) and imaginary (right) parts of the complex eigenfrequencies. The blue dashed lines indicate the lossy mode, while the red dashed lines represent the BIC mode. In **a** and **c**, the real parts are non-degenerate. When the radius is too small, the lossy mode appears above the BIC, indicating a positive *κ*. Conversely, when the radius is too large, the lossy mode appears below the BIC, indicating a negative *κ*. The size of the bandgap depends on the absolute value of *κ*. In **b**, the real parts degenerate at the Γ point, indicating a negative *κ* = 0, and characteristic branching can be observed off-Γ point, indicating the presence of EPs.

It is apparent that *ω*_eig1_ is a pure real number without radiation losses, representing the ideal FW-BIC, while *ω*_eig2_​ has a real part equal to *ω*_eig1_ but experiences the total sum of the imaginary parts of the two modes, representing a more lossy mode. The BIC can occur in either the low-frequency or high-frequency band, depending on the sign of the near-field coupling coefficient *κ*. Specifically, when *κ* is positive, the BIC appears in the lower band, whereas when *κ* is negative, it appears in the upper band. Interestingly, when *κ* = 0, the energy bands degenerate, creating partial conditions for the realization of the EP. To demonstrate this process, we illustrate the evolution of the BIC under different coupling conditions in our cases, as shown in Fig. S1.

The EP condition corresponds to the scenario where both the real and imaginary parts of the characteristic frequencies are equal, which implies that the square root term in equation (S5) equals zero. By combining this with *κ* = 0, the EP condition can be derived as

$\left| \omega_{1}-\omega_{2} \right|=2\sqrt{\gamma_{1}\gamma_{2}}$

Thus, the eigenfrequencies can be simply expressed as

$\omega_{\mathrm{eig}\_\mathrm{EP}}=\frac{(\omega_{1}+\omega_{2})-i(\gamma_{1}+\gamma_{2})}{2}$

We also present the relationship between BIC and EP under different values of in-plane wave vector calculated by TCMT, as shown in Fig. S2.


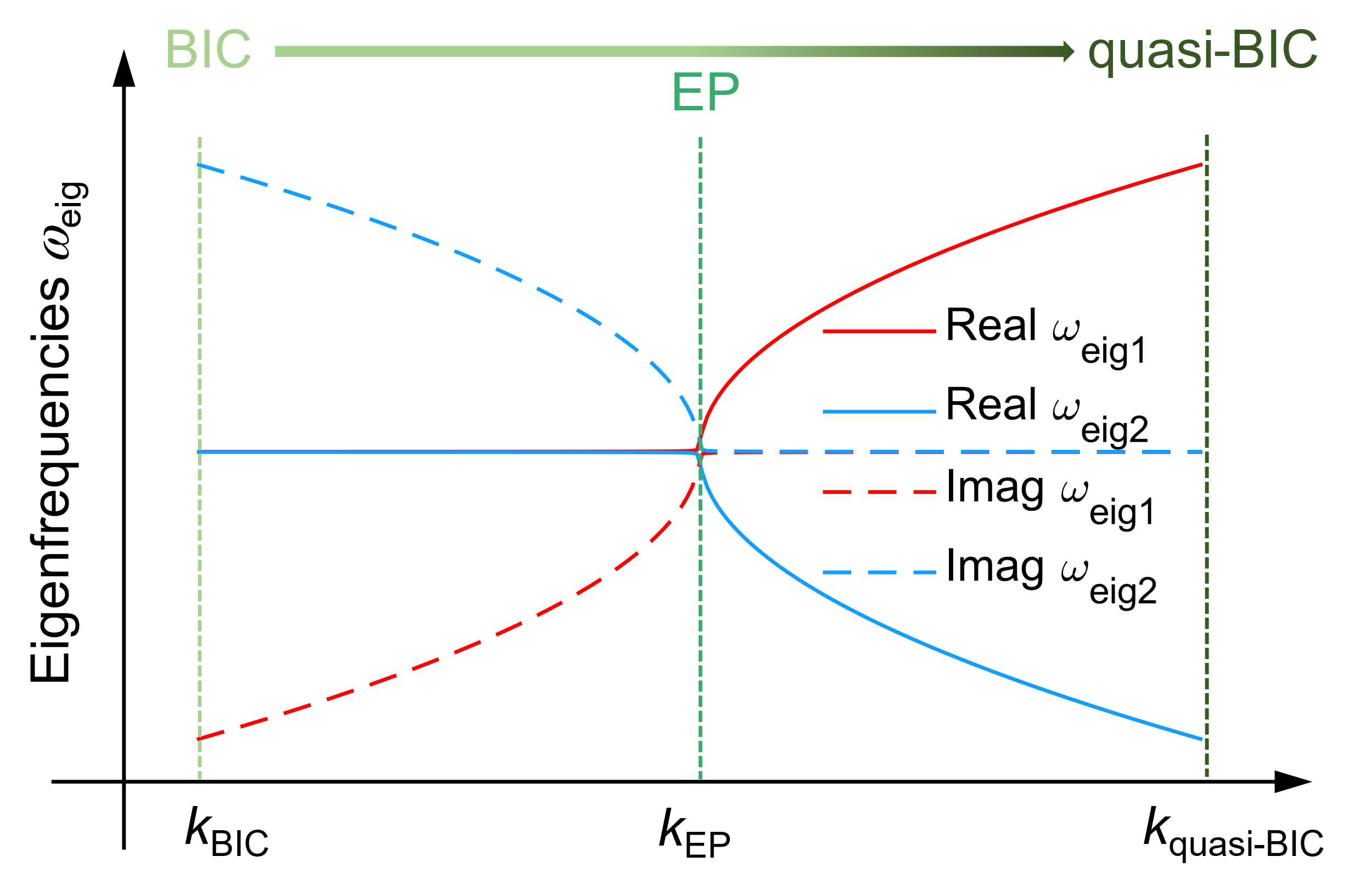


**Fig. S2.** Dependence of the complex eigenfrequencies on the in-plane wave vector. The solid red line (dashed) indicates the real (imaginary) part of mode 1, and the solid blue line (dashed) indicates the real (imaginary) part of mode 2.

In our case, we calculated the variation in the imaginary parts of the eigenfrequencies of the two modes (TE_1,2_) forming the EP at 0.56 THz, and compared them with the two isolated BIC modes at 0.47 THz (BIC_1_) and 0.67 THz (BIC_2_) in the same system, as shown in Fig. S3. It is clear that, with a small change in ***k***, the imaginary parts of TE_1_ and TE_2_ exhibit a sharp variation, which is different from the linear variation observed in the case with only BIC. Additionally, as described in the main text, the EPs derived from BICs also exhibit high *Q* factors, indicating that they inherit the unique advantages of both EPs and BICs. This holds great potential as a feasible approach for next-generation high-sensitivity sensing technologies.


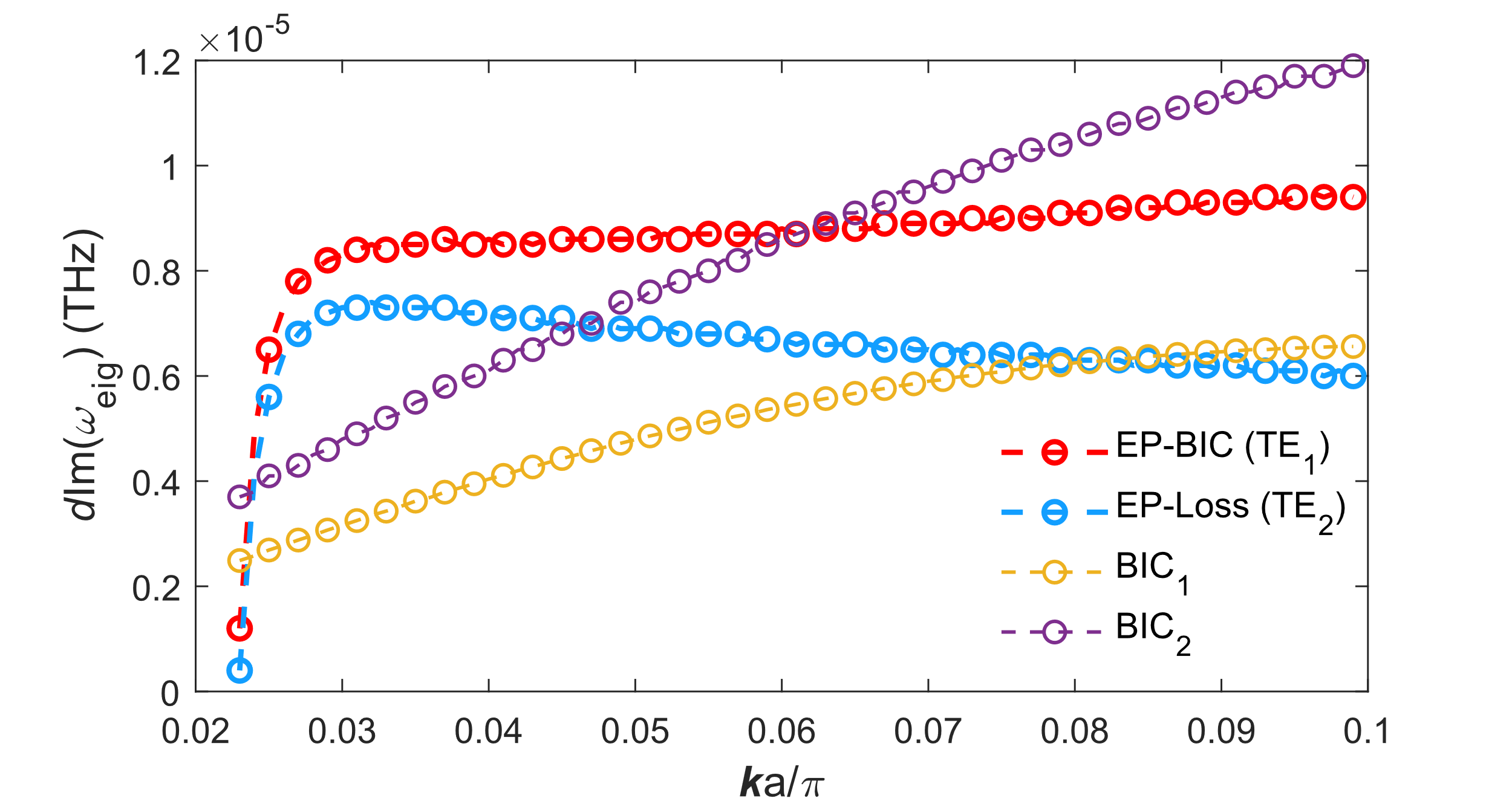


**Fig. S3.** The comparison between the BIC-derived EP and the pure BIC regarding the change in the imaginary parts of the eigenfrequencies. EP-BIC: TE_1_ mode, EP-Loss: TE_2_ mode, BIC_1_: isolated BIC modes at 0.47 THz, BIC_2_: isolated BIC modes at 0.67 THz.

**Section 2. Effect of material losses**

Considering material losses, the effective Hamiltonian can be expanded as:

$H_{\mathrm{eff}}=\left( \begin{matrix} \omega_{1} & \kappa\\ \kappa& \omega_{2} \end{matrix} \right)-i\left( \begin{matrix} \gamma_{1} & \gamma_{12} \\ \gamma_{\text{21}} & \gamma_{2} \end{matrix} \right)-i\left( \begin{matrix} \gamma_{i1} & 0 \\ 0 & \gamma_{i2} \end{matrix} \right)$

where *γ*_i1_ and *γ*_i2_ are the intrinsic material loss rates. By solving for the eigenvalues of this Hamiltonian, we obtain:

$$\omega_{\mathrm{eig}1,2}=\frac{(\omega_{1}+\omega_{2})-i(\gamma_{1}+\gamma_{\text{i1}}+\gamma_{2}+\gamma_{i2})\pm\sqrt{\left[ (\omega_{1}-\omega_{2})-i(\gamma_{1}+\gamma_{\text{i1}}-\gamma_{2}-\gamma_{i2}) \right]^{2}+4(\kappa-i\sqrt{\gamma_{1}\gamma_{2}})^{2}}}{2}$$


This form closely resembles Eq. (2) in the main text, but includes the contribution of silicon absorption. Under the condition where the discriminant of the square root term vanishes, the system reaches an exceptional point. This yields the generalized EP condition as

$$\omega_{1}-\omega_{2}=\left| 2\sqrt{\gamma_{1}\gamma_{2}} \right|$$

$\gamma_{1}+\gamma_{\text{i1}}-\gamma_{2}-\gamma_{i2}=\left| 2\kappa\right|$

Importantly, when $\gamma_{i1,i2} 0$ , the EP condition reduces smoothly to that given in the original lossless model, confirming the robustness of our results under weak material absorption.

By defining${}_{1,2}=\gamma_{1,2}+\gamma_{i1,i2}$ , we find that this expression matches our previous derivations as Eqs. S8. As a simple illustrative case, when ${}_{1}={}_{2}$, (corresponding to *κ* = 0), the system reaches an EP, while a very small *Γ*_1_ corresponds to a BIC. The presented relationship between the *Q* factor and the two modes loss ratio illustrates how the balance of modal losses governs the transition from a BIC to an EP as shown in Fig. S4. The inclusion of material loss serves as an added background dissipation in the system.


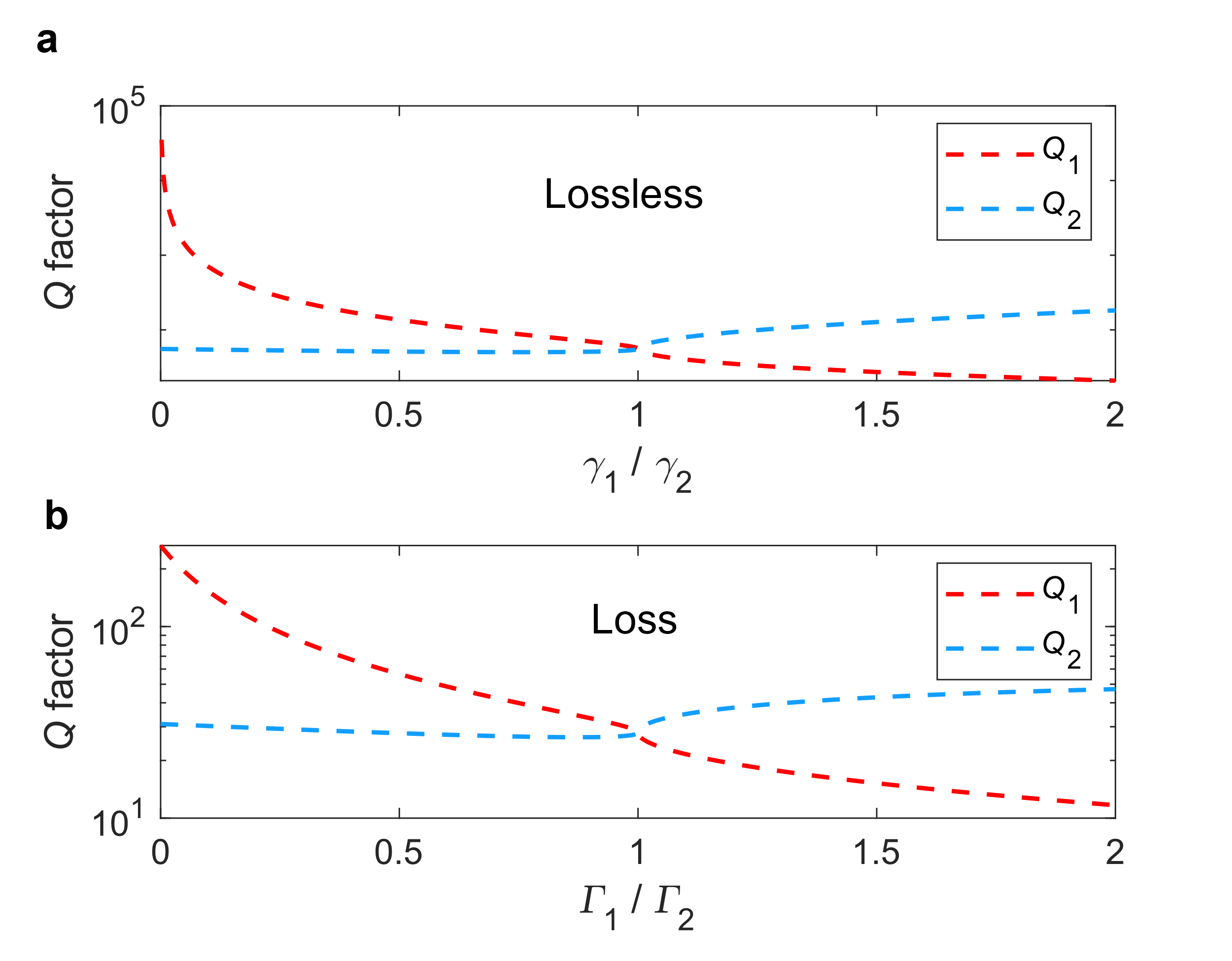


**Fig S4.** Comparison of *Q* factor decay with (**a**) and without (**b**) material loss as a function of two mode loss ratio. The resonance frequencies and loss parameters used in the calculation are: *ω*_1_ = 0.565, *ω*_2_ = 0.555, *γ*_2_ = 5 × 10⁻^3^, *γ*_i1_ = *γ*_i2_ = 5 × 10⁻^3^, with all values given in units of THz.

As a comparison, the quality factor variation with the angle for the corresponding ideal BIC mode without intrinsic material losses is shown in the fig. S5.

**
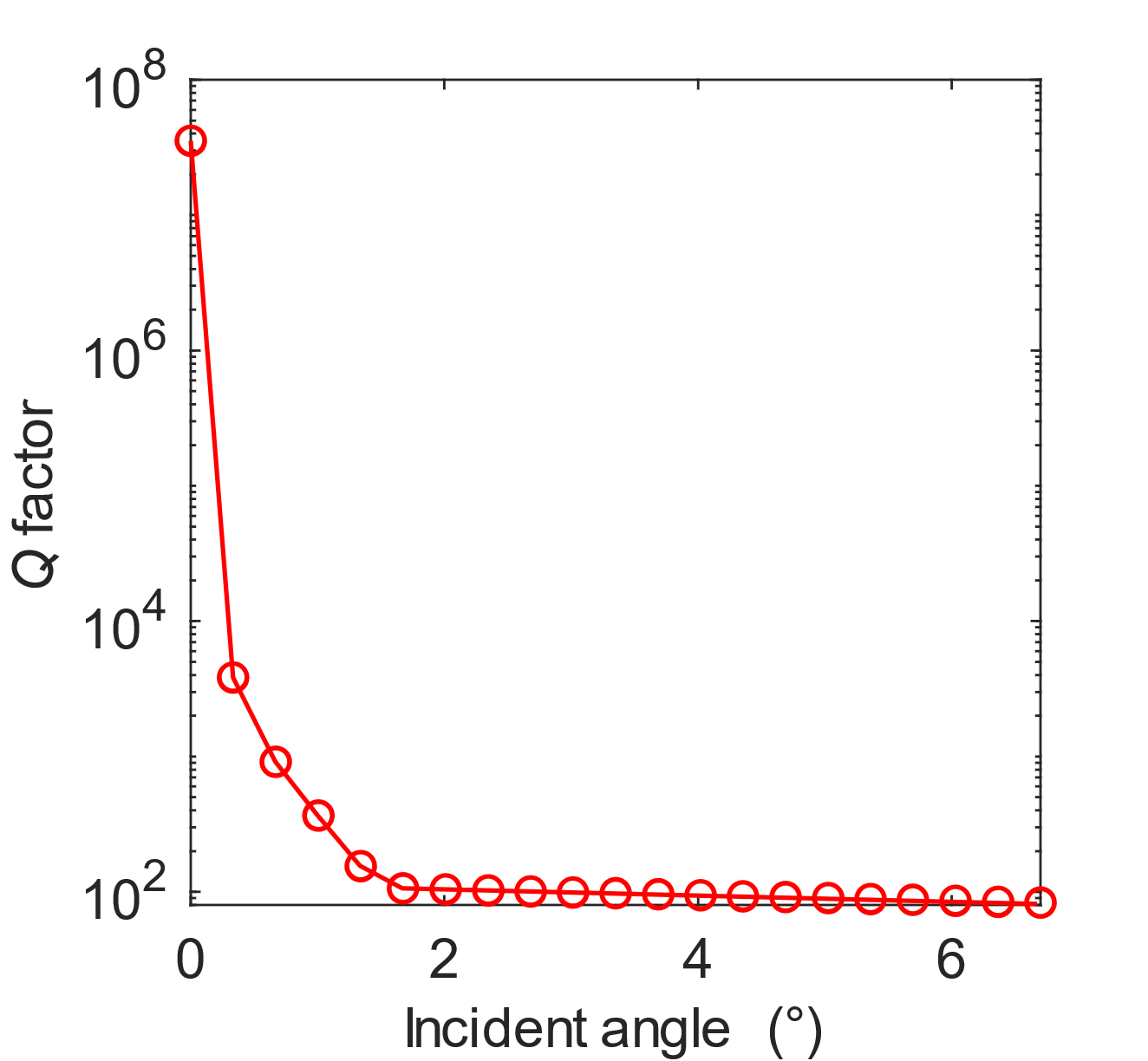
**

**Fig. S5.** Variations of *Q* factor with angle of incidence THz wave without considering material losses. The eighth power of ten can be considered infinity.

**Section 3. Extraction of eigenfrequencies from the transmission spectrum**

The transmission coefficient can be derived from Eq. S4 into the following form:

$t(\omega)=-i\frac{(\omega-\omega_{1}+i\gamma_{1}+i\gamma_{i1})\gamma_{2}+(\omega-\omega_{2}+i\gamma_{2}+i\gamma_{i2})\gamma_{1}-2\sqrt{\gamma_{1}\gamma_{2}}(\kappa-i\sqrt{\gamma_{1}\gamma_{2}})}{\left( \omega-\omega_{1}+i\gamma_{1}+i\gamma_{i1} \right)\left( \omega-\omega_{2}+i\gamma_{2}+i\gamma_{i2} \right)-(\kappa-i\sqrt{\gamma_{1}\gamma_{2}})^{2}}$

At each angle, we fitted the transmission spectrum to the TCMT expression (S11) to determine the parameters required for the eigenfrequencies in Eq. 2 of the main text. Figure S6 illustrates the comparison between the simulated transmission spectra and the TCMT-derived spectra at several representative angles. The near-perfect agreement between them validates the effectiveness of the TCMT model.


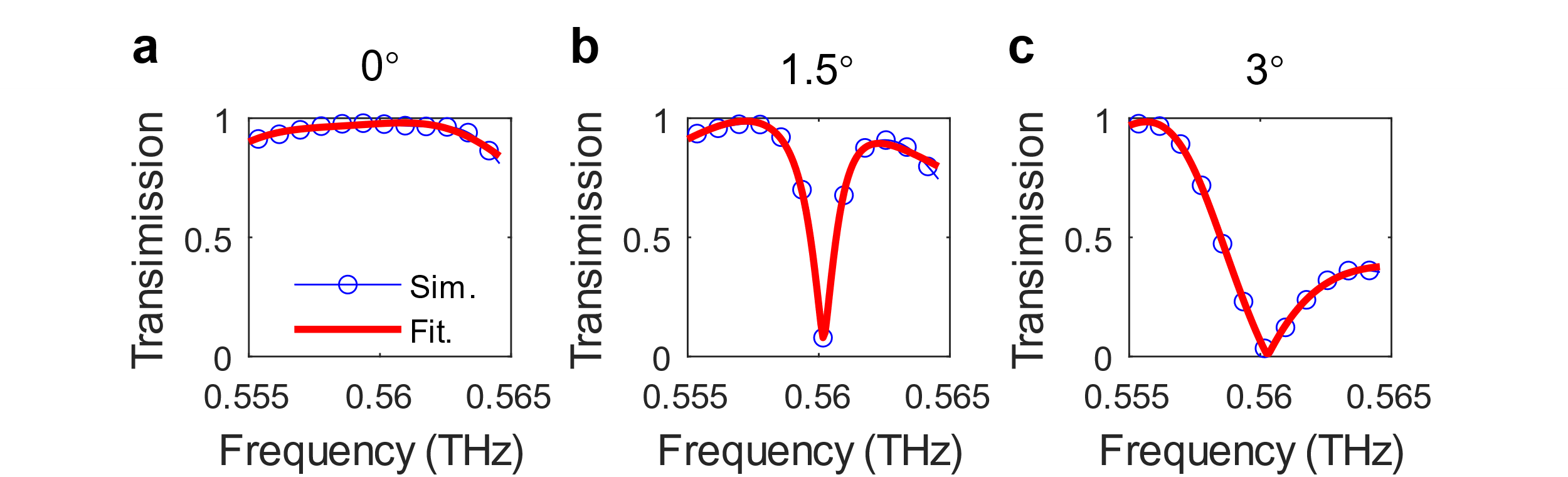


**Fig. S6.** The transmission spectra along the Γ to X direction at different incident angles (from left to right: **a**. 0°, **b**. 1.5°, and **c**. 3°) are compared with the TCMT expression in Eq. S11.

**Section 4. Calculation of far-field polarization**

The polarization vector of far-field radiation in the reciprocal space here can be defined as^2^

$c(k_{x},k_{y})=\iint_{\text{cell }} e^{ik_{x}x+ik_{y}y}\boldsymbol{E}\left( k_{x},k_{y} \right)dxdy/\iint_{\text{cell}} dxdy$

where the integration is performed in a cell sliced in the *x-y* plane at the perfectly matched layer (PML) above the metasurface. The topological charge of the BIC is defined in terms of the projected polarization vectors ***c***(*k_x_*, *k_y_*) = (*c_x_*, *c_y_*). Then, the Stokes parameter can be obtained from the following equation

$$S_{0}=|c_{x}|^{2}+|c_{y}|^{2}$$

$$S_{1} =|c_{x}|^{2}-|c_{y}|^{2}$$

$$S_{\text{2 }}=2\text{real}\left( c_{x}{c_{y}}^{*} \right)$$

$S_{3} =-2\text{imag}\left( c_{x}{c_{y}}^{*} \right)$

Along a closed and simple path in momentum space containing the BIC, the polarization vector rotates either by an integer multiple of 2π or by a negative integer multiple of 2π. The BIC is located at the vortex center of the polarization vector field, where the absence of far-field radiation implies that no far-field polarization can be assigned.

**Section 5. Sample fabrication**

The overall sample fabrication process flow chart is as described in the Methods section, and Fig. S7a provide specific details of the process as well as images of the prepared samples (Fig. S7b).

**
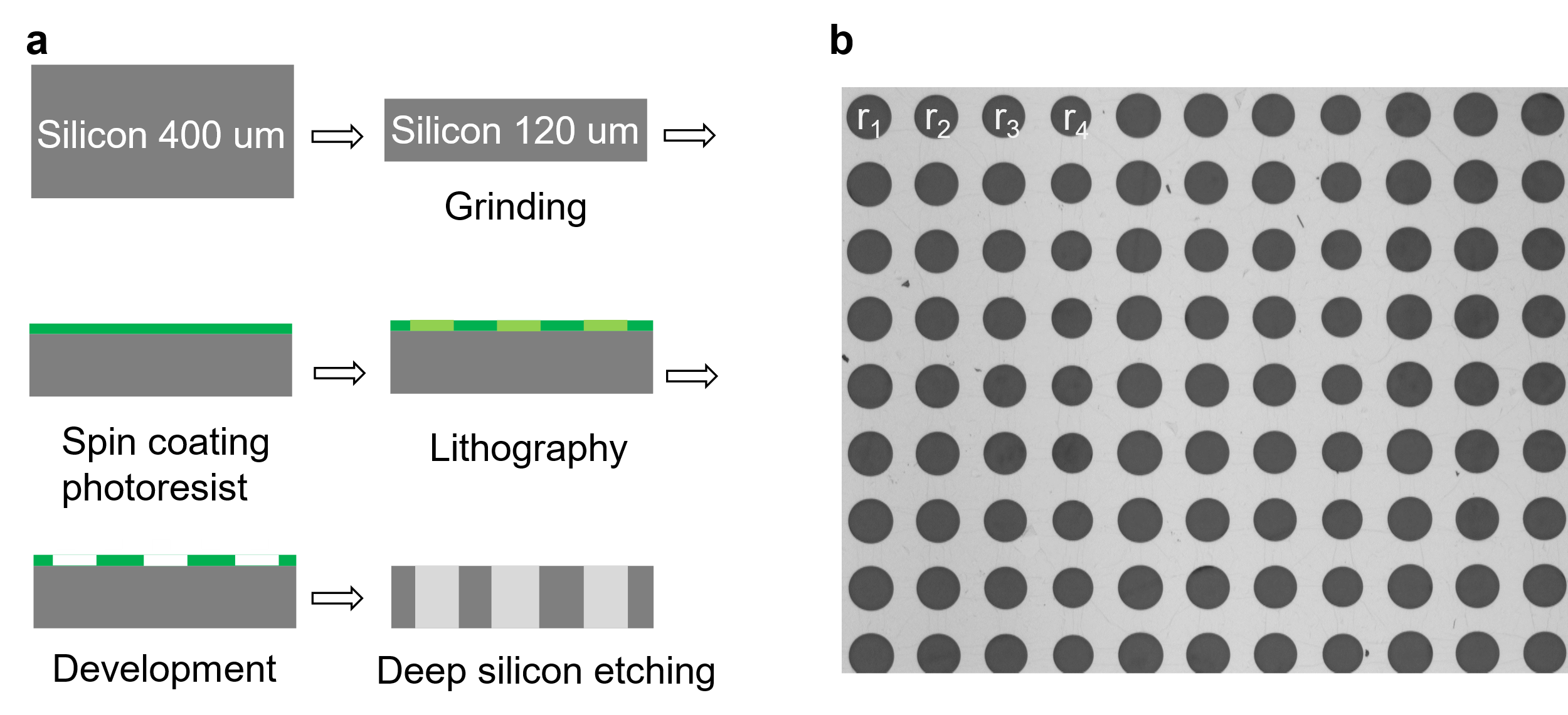
**

**Fig. S7**. Sample preparation process. **a** Flowchart of the fabrication of the proposed metasurface. **b**  Fabricated EP phase gradient metasurfaces.

**Section 6. Experimental characterization**

The response of the metasurface was characterized using THz time-domain spectroscopy (THz-TDS). Three different incident angles were selected for display, and the resulting ultra-long time-domain signal data are shown in Fig.S8a as described in the main text. Fig. S8b is a local detailed view focusing on the main THz peak.

**
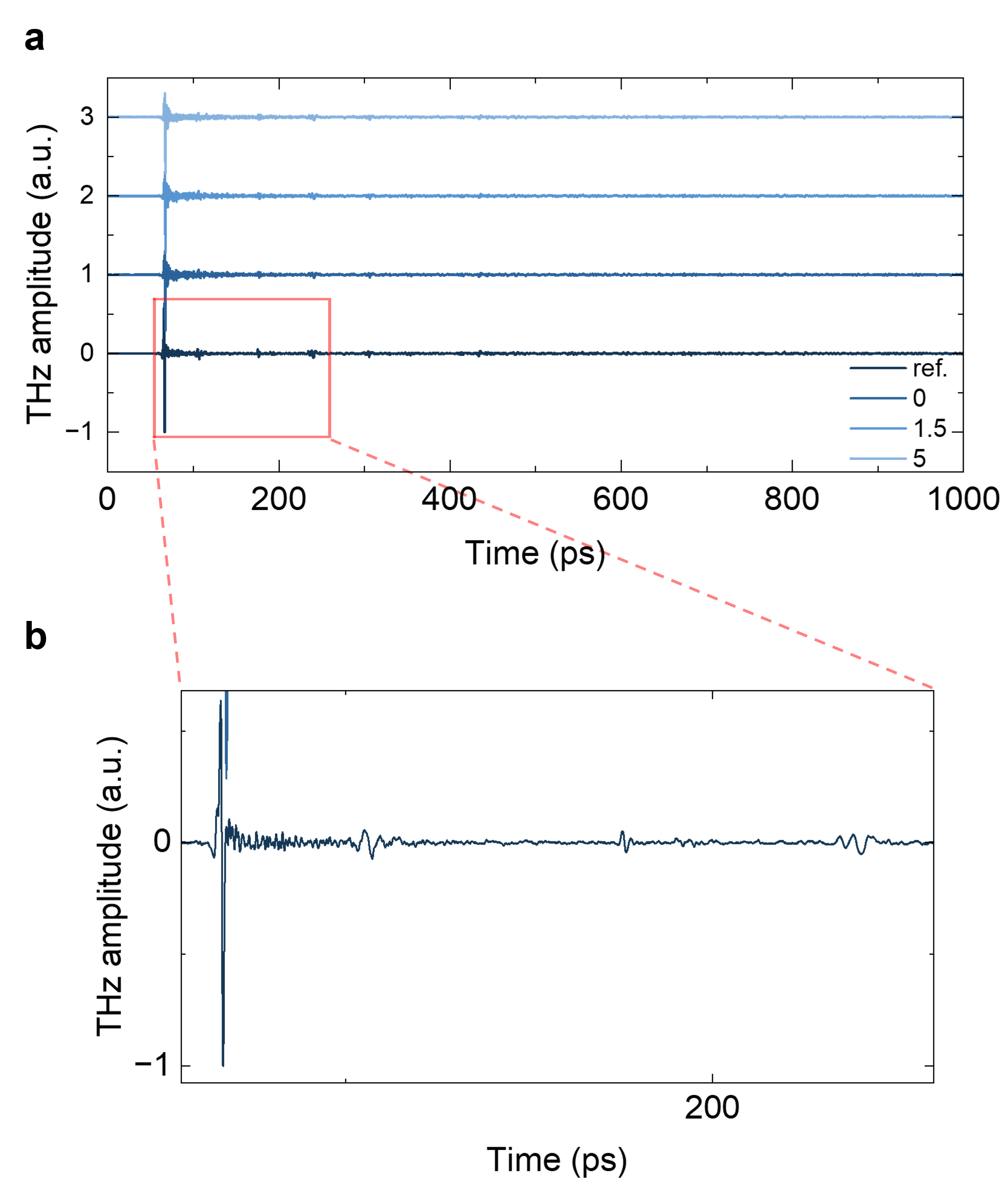
**

**Fig. S8**. Measured THz time-domain signal. **a**. Time-domain signal diagrams of the entire delay line at three selected angles and the reference. **b**. Local enlarged views.

We use the Drude model to describe the dielectric permittivity that is related to the frequency and carrier density, defined as^3^

$\varepsilon={}_{\inf}-\frac{\omega_{p}^{2}}{\omega^{2}+i{\omega\gamma}_{s}}$

where *ε*_inf_ = 11.7 is the static dielectric constant, *γ*_s_ = 1 × 10^13^ s^-1^ is the collision frequency determined by the carrier-phonon collisions in the case of low doping scattering frequency. $\omega_{p}=\sqrt{ne^{2}/0.16m_{e}\varepsilon_{0}}$is the plasma frequency, *n* is the optically induced carrier density, 0.16m_e​_ is the optical effective mass of the carriers. This model effectively captures the impact of optically induced carrier density on the dielectric constant, providing insight into how light-induced processes control the electronic states in non-Hermitian systems. Figure S9 shows the difference in the real part of the eigenfrequency at different carrier concentrations, indicating that the state of system's EP breaking is not due to frequency deviation, but rather the significant increase in material loss rate induced by optical doping.


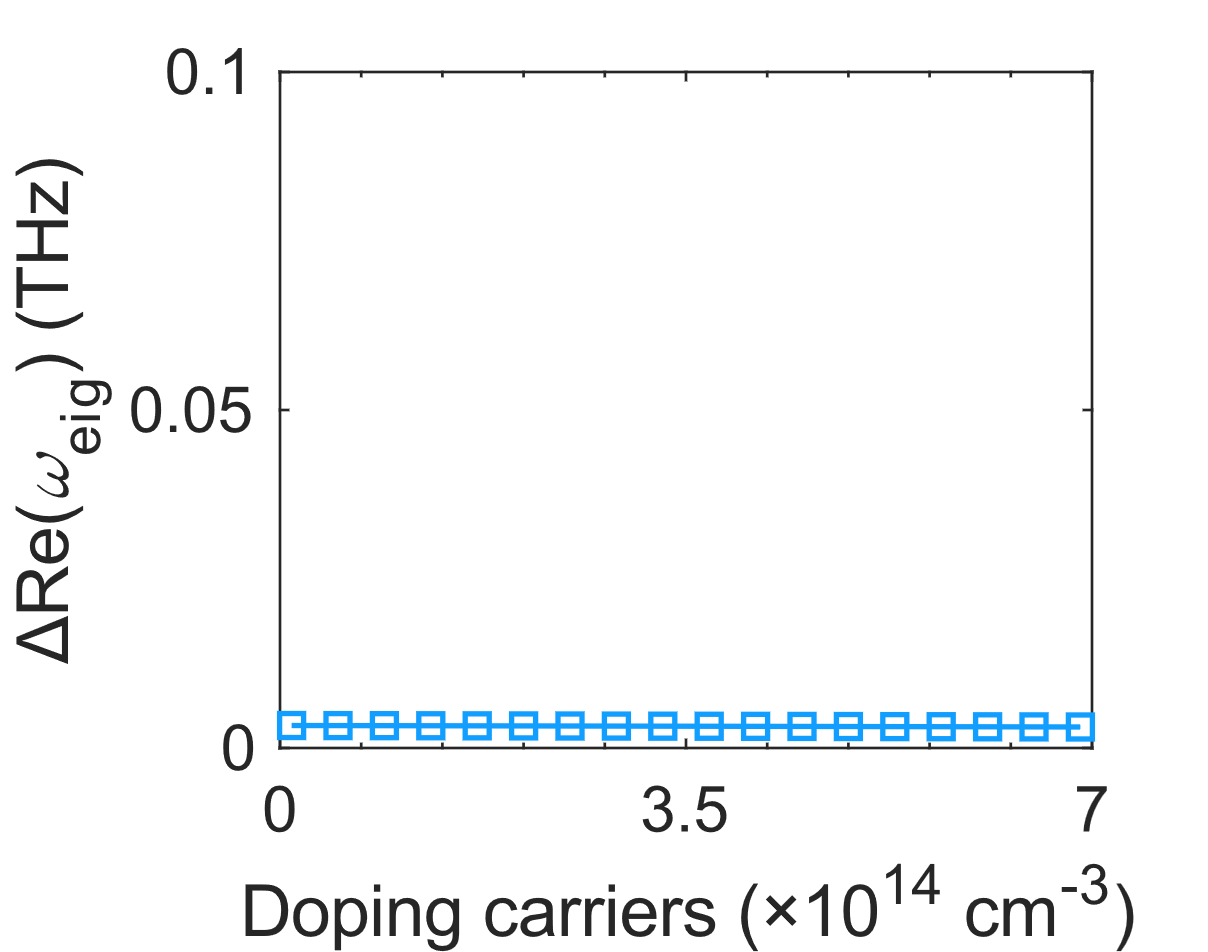


Fig. S9. The difference in the real part of the eigenfrequency at different carrier concentrations.

The carrier density introduced by photo-doping is proportional to the optical power of the pump beam. The relationship can be expressed as^4^

$n=\frac{I_{abs}\tau}{2Ad\hbar\omega}$

where *I*_abs_ ​is the absorbed power, *τ* = 25 μs is the carrier lifetime, *A* is the laser excitation area, and *d* is the penetration depth, ℏ*ω* is the photon energy of the pump laser. For the CW 980 nm laser, the penetration depth in silicon is approximately 85 μm. Hence, the transmission power can be approximated about *e*^−85/120^ ≈ 0.51. Assuming the pump beam passes through the silicon slab only once, the absorbed power is revised as *I*_abs​_ = *I*_0_​(1−*R*)(1−*T*), where *I*_0_​ is the input laser power, and *R* ≈ 32% is the reflectivity of silicon at the pump wavelength. Using an input laser power of 500 mW, the estimated carrier density is approximately about 4.92 × 10^16^ cm^−3^ which is very close to the simulation results.

**References**

1. Suh, W., Wang, Z. & Fan, S. H. Temporal coupled-mode theory and the presence of non-orthogonal modes in lossless multimode cavities. *IEEE Journal of Quantum Electronics* **40**, 1511-1518 (2004).
2. Kang, M. et al. Merging bound states in the continuum by harnessing higher-order topological charges. *Light: Science & Applications* **11**, 228 (2022).
3. Fan, K. B. et al. Phototunable dielectric Huygens' metasurfaces. *Advanced Materials* **30**, 1800278 (2018).
4. Fan, K. B., Shadrivov, I. V. & Padilla, W. J. Dynamic bound states in the continuum. *Optica* **6**, 169-173 (2019).
